# Nursing Care Plan for a Newborn with the Defect of Congenital Gastroschisis in the Postoperative Period Using ICNP^TM^ and the Dedicated Software

**DOI:** 10.3390/ijerph19063498

**Published:** 2022-03-16

**Authors:** Paulina Szydłowska-Pawlak, Olga Barszczewska, Izabela Sołtysiak, Barbara Librowska, Remigiusz Kozlowski, Per Engleseth, Michał Marczak, Dorota Kilańska

**Affiliations:** 1 Department of Coordinated Care, Medical University of Lodz, Kościuszki Street 4, 90-131 Lodz, Poland; paulina.szydlowska-pawlak@umed.lodz.pl (P.S.-P.); izabela.soltysiak@stud.umed.lodz.pl (I.S.); barbara.librowska@umed.lodz.pl (B.L.); dorota.kilanska@umed.lodz.pl (D.K.); 2 Department of Management and Logistics in Healthcare, Medical University of Lodz, Lindleya Street 6, 90-131 Lodz, Poland; michal.marczak@umed.lodz.pl; 3 Center of Security Technologies in Logistics, Faculty of Management, University of Lodz, Matejki Street 22/26, 90-237 Lodz, Poland; remigiusz.kozlowski@wz.uni.lodz.pl; 4 Narvik Campus, Tromsø School of Business and Economics, University of Tromsø, 8505 Narvik, Norway; per.engelseth@uit.no

**Keywords:** care plan, ICNP^TM^, gastroschisis, nursing care, IT, mapping terminology, classification

## Abstract

Background: Congenital defect gastroschisis manifests as a defect in the sheath in the intestine of the newborn, which is not covered by the hernia sac. In this case, the priority task of the neonatal nurse is to diagnose patient care problems quickly and accurately. Choosing the correct care plan elements has a significant impact on shortening the duration of hospitalization, reducing the number and severity of complications, and preventing their recurrence. The purpose of this study was to formulate a care plan for a newborn with diagnosed congenital defect gastroschisis in the postoperative period, using the International Classification for Nursing Practice (ICNP^TM^) within the nursing documentation and decision support system, the “ADPIECare Dorothea” software. Methods: After a review of the relevant literature and nursing documentation, a case study of a newborn with the congenital defect gastroschisis was described. A care plan was prepared using ICNP and the “ADPIECare” software. Results: It was possible to organize and standardize care plans to provide consistent and comprehensive professional nursing care. The system supporting nursing decisions suggested interventions personalized for the nursing diagnoses and to the patient needs. Conclusions: Our findings can help to optimize the nurse’s work organization to improve health care quality, outcomes, and effectiveness.

## 1. Introduction and Literature Review

Congenital defect gastroschisis involves displacement of the newborn’s intestine outside the abdominal cavity, where it is not covered by the fascia. This small defect, up to 3 cm in diameter, is usually located to the right of the correctly formed umbilical cord [1,2]. The stomach, small and large intestine, and gonads may also lie outside the abdominal cavity [2]. In utero, these organs are in contact with the amniotic fluid, which can irritate them and cause them to shorten or swell [3]. Congenital defect gastroschisis is differentiated from umbilical hernia by the absence of a hernia sac limiting the viscera [3]. According to the literature, gastroschisis occurs in 1 in 4000 live births [1]. The majority of pregnancies complicated by gastroschisis are diagnosed prenatally [4]. The incidence of selected birth defects (per 10,000 births) in years 2011–2015 was 641 [5].

The priority goal of treating congenital defect gastroschisis is to place the intestines into the abdominal cavity of the newborn as soon as possible followed by complete reconstruction of the hernial sac. Ideally, a one-step operation should be performed immediately after birth. The choice of surgical treatment option depends on the volume of displaced viscera, local bowel condition, and the size of the abdominal cavity [2].

In cases where a one-step procedure is not feasible, a multi-stage procedure is recommended: The intestines are temporarily covered using plastics (e.g., silastic, tegmentum) (silo), with abdominal inclusion only performed using skin, without abdominoplasty [2].

The temporary stitching of the silo coating requires the silo bag to be hung above the newborn. The capacity of the abdominal cavity is gradually increased using gravity and by shrinking the bag. As a consequence, the intestines and organs return to the abdomen within 5–10 days [4]. This method requires the prolonged mechanical ventilation of the newborn and is associated with an increased risk of infection, including pneumonia or wound infection [6]. In addition, multi-stage treatment is associated with a number of care problems deriving from intestinal fistulas, multiple general anesthesias, delayed introduction of parenteral nutrition, and an extended period of hospitalization [7].

The significant role in newborn care, as part of therapeutic team, belongs to nurses. They focus on diagnostics, treatment, and nursing, adjusting to the individual needs of a patient [8]. The priority task of a neonatal nurse is to perform a quick and accurate diagnosis of patient care problems. Choosing the correct care plan elements can make a significant contribution to shortening the duration of hospitalization [7], reducing the number and severity of adverse events, and preventing further complications such as necrotizing enterocolitis (NEC) [9]. Nursing care plans can help with patient recovery, and using nursing referenced terminology in care plans will help nurses to achieve the care effectiveness, which could be compared and standardized [10].

The purpose of this study was to demonstrate the formulation of a plan of nursing care for a newborn diagnosed with the congenital defect of gastroschisis in the postoperative period with using terms from the International Classification for Nursing Practice (ICNP), which was included in the “ADPIECare Dorothea” software.

### The “ADPIECare Dorothea” Software Description—Application of IT Systems in Care Planning

The “ADPIECare Dorothea” software is the tool that allows the nurse or student to document nursing care plans with nursing standardized terminology—ICNP terminology, which is recommended by the International Council of Nurses [10,11] and United Nations for nursing statistics [12]. The ICNP was implemented in the software for a nursing assessment tool using the terms of ICNP from the Focus Axis and for building the care plans using ICNP diagnosis, Localization, Time, and Client. The software supports decision making with the choosing of care plans from the catalogue available in the system [13]. A student or nurse can use a reference terminology in the practice to describe patient’s needs, signs, symptoms, and problems in accordance with the recommendation of the Ministry of Health [14,15,16]. The usage of HL7 CDA standard in the ADPIECare system helps to assess the effectiveness of nursing care using sensitive nursing indicators [17,18].

**Figure 1:**
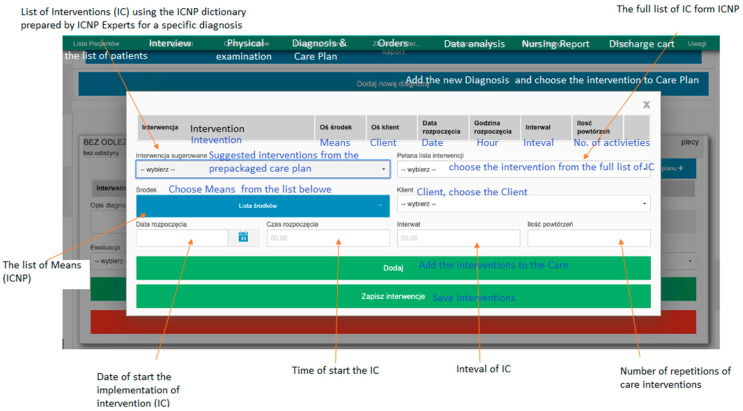
ICNP clinical support system “ADPIECare Dorothea” with care plan window, Available online: http://ciitt.umed.pl/system-dokumentacji-i-wsparcia-prac-pielegniarskich-adpiecare-dorothea/ (accessed on 9 January 2022).

There is interest in a number of countries in ICNP as a standard terminology, which is recommended internationally [11]. It represents a practical standard for describing and documenting nursing care, especially in the field of nursing diagnoses (DC), nursing interventions (IC), and the results of care expressed in a reformulated diagnosis within ICNP [16]. Attention was given to special nursing problems occurring in the postoperative period in newborns with an implanted silo sac and after the corrective procedure. Nurses can use software to prepare care plans with selected interventions. The recommended interventions were prepared by the nursing experts specially for the software ADPIECare, which we can see in Figure 1.

**Figure 2:**
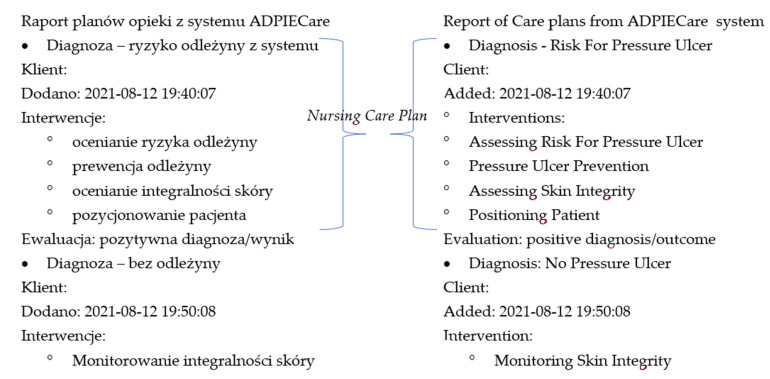
The nursing care plan report from the ADPIECare system, Availaible online http://ciitt.umed.pl/system-dokumentacji-i-wsparcia-prac-pielegniarskich-adpiecare-dorothea/ (accessed on 9 January 2022).

The system helps users prepare reports of the nurse care plans, which can be sent in during the Transition of Care process to another setting for continuity of care. Figure 2 shows the report of nurses care plan “Risk of pleasure ulcer”, which was made in the ADPIECare system.

The use of the application containing a dictionary allowed users to draw attention to the need for completeness of describing the patient. The limitation of the use of the application is its lack of availability in practice; hence, the case description was transferred from paper documentation to the system for the purposes of publication. It can be difficult to use the dictionary itself by nurses who do not know beforehand how to document patient information. Preparing a tool for nurses that will not only be a place to save data but also become a system prompting a solution for care planning seems to be key to facilitating care planning. The documentation of care plans should be implemented to the IT system, which may support nurses in administrative tasks and shorten their documentation time, which according to the scientific research may sometimes amount to 50% of a nurse’s working time [19,20]. Then, the nurse can focus more on planning interventions that will be effective for this group of patients. It will also improve the quality of documentation, thus increasing patient safety.

## 2. Materials and Methods

**Figure 3:**
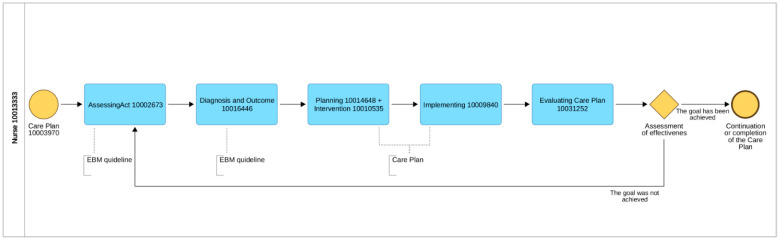
BPMN process of nursing care plan with using ICNP (compiled by Paulina Szydłowska–Pawlak, ADONIS, UMed. 2022).

A single case study was carried out to achieve the necessary detail required in this type of scientific observation. This includes applying a research strategy that involves multi-methods such as observations, interviews, and secondary data to study a real-life phenomenon in its natural setting [21]. The care plans were prepared on the basis of the 15 days of observation. This observation was recorded in the nursing paper documentation. The nursing paper documentation was analyzed by the students, and the experts from the department and then from the ICNP center were consulted. The nursing care plan elements (diagnoses, interventions with means, and evaluation outcomes) in this case were created by using the International Classification for Nursing Practice (ICNP) with the ICNP experts from Accredited Centre R&D at Medical University of Lodz. In the first step, the identification and validation of terms related to relevant altered needs for the case with the congenital defect of gastroschisis in the postoperative period was made. Then, cross-mapping of altered needs with ICNP 2019 version Focus Axis terms [22] was made by the team of experts. As a result, we identified the nursing language terms related to caring for this case, which was used to construct nursing diagnosis, outcome, and intervention statements following the recommendations of ICN and ISO 18.104: 2014. In the first step, the team prepared care plans on paper in accordance with ICN recommendations [23]. The construction of the statements of nursing diagnoses, interventions, and outcomes/diagnoses was carried out. The nursing process is a continuous repetitive circle (continuous model of care) in which the care plan begins from the first step, which is diagnosis as the result of assessing and ending another diagnosis, which is the result of evaluation and starting another care plan and at the same time is the outcome of the effectiveness of care. In each step of the nursing process, the nurse should use references terminology, which was recommended for nursing practice—ICNP. This is the main issue related to recognizing nursing impact on health care and patient outcomes. The steps of the nursing process are described in Figure 3 below.

For each diagnosis, Focus Axis terms were used, and other terms from ICNP were added depending on the need and specificity of Judgment, Client, Location, and Time axes. For each Intervention, an Action axis term and a Client term were used, which were considered as a term for any of the axes except Judgment [24]. It was also considered the ISO 18.104: 2014—Health Informatics: categorical structures for the representation of nursing diagnoses and nursing actions in terminology systems, in which a nursing diagnosis can consist of a single Focus term along with an axis at the end of the Judgment axis or a clinical finding [24], and in the next step, the care plans were prepared in the ADPIECare software [25].

The nursing care plan was structured by using the ADPIECare nursing decision support system (DSS). This is the first Polish system to enable the construction of individual nursing care plans using ICNP. It sets nursing diagnoses with using primary terms from different axes, selects actions tailored to a specific disease entity, and also helps to assess the nursing care provided [18,26]. The ADPIECare was developed in 2016 by using the Nursing International Minimum Data Set (I-NMDS) in nursing, which is recommended for statistics in the nursing practice [12].

The IT system is the tool that supports describing patients’ needs, symptoms, and will help to better recognize the diagnosis that nurses can choose to describe patient status of care. More details are described below.

## 3. Case Study Description and Outcomes

### 3.1. Case Study Description

The newborn, a female, was delivered by caesarean section at 38 WGA (Weeks Gestational Age) with a weight of 2450 g. The congenital defect of gastroschisis had been diagnosed at 14 weeks of gestation during prenatal screening. The newborn was born in a 3rd level specialist reference healthcare center. The exposed intestines were secured on the delivery table, and these plus the lower parts of the body were covered with a plastic bag. The newborn was transferred to a pediatric surgery team. The exposed bowels were replaced in the child’s abdominal cavity in a multi-stage manner using a silastic bag (silo).

One week after the silo was put on, the intestines had returned to the abdominal cavity. Then, the silo was removed, and the anterior abdominal wall defect was surgically closed using biological material.

After each surgery, the newborn was admitted to the Intensive Care Unit where it was intubated and mechanically ventilated. A catheter for urine was inserted. A central venous line was inserted for parenteral nutrition and a peripheral vein was also accessed. An arterial catheter was inserted for measurement of blood pressure and pulse. Vital signs were monitored.

### 3.2. Case Study Outcomes

The proposed nursing care plan for the postoperative period of a newborn with congenital defect gastroschisis repair included nine nursing diagnoses along with a number of potential diagnoses: pain caused by a wound, parental stress, risk of infection (associated with a surgical wound, central, peripheral and arterial cannula maintenance, bladder catheter maintenance), risk of pressure sores, risk of respiratory dysfunction, risk of impaired nutritional status, and risk of disturbed vascular process in the abdomen or abdominal wall.

Interventions that were used for nursing care for the postoperative period of a newborn with congenital defect gastroschisis repair in this ward included care plans that were described in natural language. In these care plans, nurses’ constant and non-constant terms were used: monitoring of the support dressing for the amount and quality of secretions; monitoring the intestines in the dressing; assessing their color, degree of hydration, size, and the process of intestinal drainage; care and monitoring of the stomach decompression probe; assessing the quantity, type and color of the stomach content; and monitoring swelling at the location [7]. In the event of a full correction of the defect, with closure of the abdominal wall, the postoperative wound should be monitored in the care plan according to the protocol, with careful observation of abdominal wall tension, drawing of venous vessels on the abdominal wall, and measurement of the abdominal circumference at the navel line.

The nursing care plan includes assessing the potential for the newborn to receive breast milk from the mother. Minimal trophic nutrition usually starts with 2 mL, and it is extremely important to give the newborn breast milk if possible. The nurse’s task is to support and educate the newborn’s mother regarding stimulating lactation, expressing breast milk and storing the breast milk properly.

For the purpose of defining the groups of diagnoses for the case study, the scheme proposed by the ICN [27], da Silva et al. [28], and additionally the ICNP Catalog “Community Nursing”—Nursing Minimum Data Set [29] with classification for aggregated nurses’ diagnosis and interventions for caregivers were used. The result is shown in Table 1.

In the presented summary of care plans, nine diagnoses were made, eight of them related to Body Process Diagnosis and Outcome, and one related to Psychological Process Diagnosis and Outcome. In the case of a child, seven plans concerned potential diagnoses, one concerned current diagnosis. In one, the care plan concerned a parent-current diagnosis. In total, 111 terms from the ICNP international vocabulary have been assigned to the care plan. Nine diagnoses were made, and the diagnoses were completed with terms from the location axis—a total of seven terms. Appropriate interventions were selected for each diagnosis, and a total of 58 terms from the IC axis were selected. Some interventions have been completed with terms from the subject axis: six terms in total. Each intervention has a timeline from the measures axis. A total of 22 terms were used, which were repeated many times, resulting in a total of 103 terms. The most common term in the axis of measures is Nurse [10013333], which has been used in interventions for each diagnosis presented in the paper. Another term, the Assessment tool [10002832], has been used in eight out of nine diagnoses. The Aseptic technique [10002639] was used with the interventions proposed for four diagnoses; Antibiotic [10002383], Physician [10014522], and the Medication administration technique [10006322] were assigned to three diagnoses, and the remaining agents (M) were used only once. An evaluation was performed for each care plan, resulting in a further nine diagnoses/outcomes.No diagnosis was assigned for Elimination, Sleep and Rest, Exercise and Physical Activities, Body Care, Therapy, Safety, Love, Communication, Sociability, Space, Self-Image, and Spirituality. No interventions have been assigned to Hydration, Sleep and Rest, Exercise and Physical Activities, Thermal Regulation, Neurological Regulation, Safety, Gregariousness and Self-Image.

## 4. Discussion

The usefulness of the ICNP^®^ in nursing practice was recognized worldwide [30]. One of the ICNP subsets is the prenatal nursing care catalogue. The literature suggests that usage of the subset in the electronic health record (EHR) might improve prenatal care by reducing the use of paper-based documentation, ameliorating communication, easing the documentation workload, and optimizing storage utilization [31]. A Taiwanese study showed that 87% of clinical nursing problems, and as a consequence nursing care plans, could be cross-matched with ICNP [32]. Another article stated that clinic care classification systems are time consuming and slow; however, they provide better support to the clinical nursing information system [33].

To fully explore those benefits, the broad implementation of the ICNP in combination with e-tools is required [34]. An example of software supporting the decision-making process in nursing is Unified Modeling Language (UML). This software writes language known to precisely represent the perspective of the end-users. In the study of Choi et al., the software was used to translate a guideline for medication management in elderlies encoded with the ICNP. The authors emphasized that such a solution may facilitate cooperation between a nursing informatician and a developer in order to implement a computer system that is preferred by nurses, and it may ensure that the model is easily maintained [35]. The literature emphasizes that the proper terminology system should include broad ranges of semantic relations and well-programmed systematic mapping between the model of a terminology and an information model [36].

Some ways to facilitate the implementation of the ICNP with e-tools are the establishment of strategic governmental decisions, reinforcement of collaboration between governments and IT companies [34], and improved validation of a nursing practice [37,38].

The presented case study concerned a topic and specificity that had not been discussed before. Therefore, it is impossible to directly compare the results of the study with the results of other studies. However, a case study similar to that presented in this article was found. It analyzed a premature baby. The study has shown that a professional nursing care plan is very important. The care should be based on monitoring, observation, and parental involvement in the daily care of the child [39]. The engaged nurse facilitates communication between the baby, family, and the surrounding system [40]. Treatment pathways are well-recognized tools that result in a better outcome of delivered care. One of the examples is the Vegunta et al. [41] study where the authors established a clinical pathway in order to achieve optimal management after antenatal diagnosis of gastroschisis. The study was conducted on 30 patients. The new protocol consisted of an elective cesarean section and early gastroschisis repair. The results showed that primary repair was achieved in a higher proportion, and the duration of mechanical ventilation and length of stay were shorter. The Picado et al. study [42], based on happy breastfeeding analysis, adds that standardized work is beneficial for the decision making and performance of nursing staff. Research should discuss the results and how they can be interpreted from the perspective of previous studies and of the working hypotheses. The findings and their implications should be discussed in the broadest context possible. Future research directions may also be highlighted.

The limitation of the ADPIECare system is the use of the ICNP dictionary, which is not commonly taught in nursing in Poland; hence, it can make care planning difficult. It can be challenging to use the dictionary itself by nurses who do not know beforehand how to document patient information. Our own research showed that users who had no contact with the classification documented care plans for a longer period of time. The authors presented the catalog “Catalog of Prenatal Nursing Care” to indicate that it is very easy to build a plan and show the results of care. It is the only ICN catalog that shows all elements of the nursing work process. It was chosen as the directory most closely related to neonatal care. Additionally, since the tools are new, education and awareness must be raised among healthcare professionals.

## 5. Conclusions

The goal for nursing care for a newborn with congenital defect gastroschisis repair is to have a comprehensive, systematic, and continuous care process. In postoperative care, the nurse identifies diagnoses and therapeutic nursing care. Such comprehensive nursing care is based on the nurse’s specialist knowledge. All care provision activities are undertaken as a response to the patient’s condition and all the signs and symptoms. In this way, the newborn will remain stable while recovering and healing in a forced position [43].

The nurse’s priority in postoperative care is to detect early complications of surgery and mechanical ventilation. Therefore, it is extremely important to observe the general condition of the patient and monitor all vital signs.

The nursing care plan for the newborn described in this case study used the ICNP terminology and ADPIECare documentation and clinical decision support system to organize and standardize diagnoses, interventions, and outcomes. These tools made it possible to formulate nursing diagnoses and potential diagnoses and supported planned interventions and the continuous documentation of results [44]. The software, documentation, and decision support system for nursing practice was found to be a valuable part of the decision-making process and transparency of the nursing care [13,44,45]. The system suggested individual interventions that were personalized to the patient (Ryc. 1) and includes ready-made care plans, according to patient needs. Care plans were prepared according to ICN catalogues, which were validated by nurses experts. In the application, we also can summarize care plans, interventions, and evaluate the effectiveness of the work or the workload of nurses. Such data can help optimize work organization to achieve high levels of health care and patient safety. The team of experts evaluate three different categories of nursing diagnoses according to the described case study. The terms mapped from nursing records to the ICNP show that diagnoses and interventions have not been identified in many areas of nursing care. This may suggest that the documentation of observations is omitted, which would allow for a more comprehensive presentation of the problems of caring for this type of patients. Using a nursing classification in the app can help you record more accurate observations, including those that are not routinely documented.

Introducing classification into practice, as proposed through this analysis, would help identify diagnoses and interventions that were not identified in the analysis of natural language documentation.

## Figures and Tables

**Table 1:** Nursing care plan for a newborn in the postoperative period with diagnosed congenital defect—gastroschisis.

**Nursing Diagnosis and Results**	
**Human Psychobiological Needs**	
Oxygenation	Risk of impaired respiratory system function [10037346]
	Effective respiratory status [10033830]
Hydration	N/A
Nutrition	Risk of impaired nutritional status [10037224]
	Positive nutritional status [10025002]
Elimination	N/A
Sleep and Rest	N/A
Exercise and Physical Activities	N/A
Body Care	N/A
Cutaneous-Mucous Integrity	Risk for pressure ulcer [10027337]
	No pressure ulcer [10029065]
Physical Integrity	Risk of infection [10015133]
	No infection [10028945]
	Symptom control [10025820]
Thermal Regulation	N/A
Neurological Regulation	N/A
Vascular Regulation	Risk of hemorrhaging [10017268
	Positive vascular process [10028118]
Therapy	N/A
**Human Psychosocial Needs**	
Carers	Parental stress [10001385]
	Decreased stress [10027929]
Safety	N/A
Love	N/A
Communication	N/A
Gregariousness	N/A
Space	N/A
Self-Image	N/A
Human Psychospiritual Needs	N/A
Spirituality	N/A
**Nursing Interventions**	
**Human Psychobiological Needs**	
Oxygenation	Maintaining ventilation with a mechanical ventilator [10046258]
	Maintaining airway clearance [10037351]
	Evaluating respiratory status after operation [10007169]
	Suctioning the airway [10044890]
	Monitoring respiratory status [10012196]
	Assessing respiratory status using monitoring device [10002799]
Hydration	N/A
Nutrition	Collaborating on dietary regime [10026190]
	Assessing risk for impaired nutritional status [10040921]
	Weighing patient [10033323]
	Monitoring weight [10032121]
	Monitoring nutrition [10036032]
	Managing vomiting [10046329]
	Feeding patient [10046150]
	Promoting exclusive breastfeeding [10039437]
	Assessing nutritional status [10030660]
	Monitoring bowel motility [10037211]
Elimination	Urinary catheter care [10033277]
	Measuring fluid output [10039250]
	Assessing urinary system [10002866]
Sleep and Rest	N/A
Exercise and Physical Activities	N/A
Body Care	Perineal care [10045154]
Cutaneous-Mucous Integrity	Monitoring wound healing [10042936]
	Surgical wound care [10032863]
	Assessing risk for pressure ulcer [10030710]
	Pressure ulcer prevention [10040224]
	Positioning patient [10014761]
	Skin care [10032757]
	Invasive device site care [10031592]
Physical Integrity	Monitoring signs and symptoms of infection [10012203]
	Assessing exposure to contagion [10044013]
	Disinfecting [10006044]
	Continuous surveillance [10005093]
	Preventing infection [10036916]
	Monitoring laboratory result [10032099]
	Collecting specimen [10004588]
Thermal Regulation	N/A
Neurological Regulation	N/A
Vascular Regulation	Maintaining intravenous access [10036577]
	Managing central line [10031724]
	Measuring radial pulse [10044740]
Pain Perception	Assessing pain [10026119]
	Monitoring pain [10038929] Administering pain medication [10023084]
	Monitoring response to treatment [10032109]
	Implementing pain guideline [10009872]
Therapy	Administering prophylactic treatment [10001827]
	Collaborating with interprofessional team on wound care [10043995]
	Administering antibiotic [10030383]
	Teaching family about treatment regime [10024656]
**Human Psychosocial Needs**	
Carers	Assessing stress level [10043809]
Safety	N/A
Love	Supporting family [10032844]
Communication	Collaborating with family [10035887]
	Teaching about caregiver child attachment [10036842]
Gregariousness	N/A
Space	Providing emotional support [1002705]
Self-Image	N/A
**Human Psychospiritual Needs**	
Spirituality	Providing spiritual support [10027067]

## Data Availability

The data presented in this study are available on request from the corresponding author.
